# The efficacy and safety of luteal phase support with progesterone following ovarian stimulation and intrauterine insemination: A systematic review and meta-analysis

**DOI:** 10.3389/fendo.2022.960393

**Published:** 2022-09-02

**Authors:** G. Casarramona, T. Lalmahomed, CHC. Lemmen, MJC. Eijkemans, FJM. Broekmans, AEP. Cantineau, KCE. Drechsel

**Affiliations:** ^1^ Department of Reproductive Medicine, University Medical Center Utrecht, Utrecht, Netherlands; ^2^ University Medical Center Utrecht, Utrecht University, Utrecht, Netherlands; ^3^ Julius Center for Health Sciences and Primary Care, University Medical Center Utrecht, Utrecht University, Utrecht, Netherlands; ^4^ Department of Obstetrics and Gynecology, University Medical Centre Groningen, University of Groningen, Groningen, Netherlands

**Keywords:** infertility, intrauterine insemination, luteal (phase) support, meta-analysis, ovarian stimulation, progesterone

## Abstract

The aim of this systematic review and meta-analysis was to update the current evidence for the efficacy and safety of progesterone luteal phase support (LPS) following ovarian stimulation and intrauterine insemination treatment (OS-IUI) for unexplained or mild male infertility. Four additional studies were identified compared to the previous review in 2017. Twelve RCTs (2631 patients, 3262 cycles) met full inclusion criteria. Results from quantitative synthesis suggest that progesterone LPS after OS-IUI leads to higher live birth (RR 1.38, 95%CI [1.09, 1.74]; 7 RCTs, n=1748) and clinical pregnancy rates (RR 1.38, 95% CI [1.21, 1.59]; 11 RCTs, n=2163) than no LPS or placebo. This effect is specifically present in protocols using gonadotropins for OS-IUI (RR 1.41, 95%CI [1.17, 1.71]; 7 RCTs, n=1114), and unclear in protocols involving clomiphene citrate (RR 1.01, 95% CI [0.05, 18.94]; 2 RCTs, n=138). We found no effect of progesterone LPS on multiple pregnancy or miscarriage rates. No correlation between drug-dosage or duration of treatment and effect size was seen. Though our results suggest both benefit and safety of progesterone LPS in OS-IUI, evidence is of low to moderate quality and additional well-powered trials are still mandatory to confirm our findings and justify implementation in daily practice.

**Systematic Review Registration:**

https://www.crd.york.ac.uk/prospero/display_record.php?RecordID=292325, identifier CRD42021292325.

## Introduction

Intrauterine insemination (IUI), combined with ovarian stimulation (OS), is a widely used treatment for unexplained or mild male infertility (1). Ovarian stimulation with low doses of gonadotropins, aromatase inhibitors (e.g., letrozole), or clomiphene citrate (CC), is generally used to achieve growth of two dominant follicles. A single bolus injection of human chorionic gonadotropin (hCG) is then given as an exogenous trigger to complete oocyte maturation and induce ovulation. Pre-washed semen is injected into the uterus at approximately 36 hours after the hCG trigger for an optimal chance of fertilization (2).

During the natural menstrual cycle, follicle stimulating hormone (FSH) promotes growth and maturation of a dominant follicle in one of the ovaries. Estrogen levels gradually build up, and during the final rapid increase, feedback mechanisms elicit a surge of luteinizing hormone (LH) and FSH secretion along with a small increase in progesterone production by granulosa cells (3). The midcycle gonadotropin surge induces both the final maturation of the oocyte and ovulation, as well as the transition of the steroidogenesis in the granulosa cells into synthesis of both estradiol and progesterone. This process of granulosa cell luteinization marks the start of the luteal phase. Driven by intensified pulsatile LH release from the pituitary, the corpus luteum produces progesterone, which allows endometrial secretory transformation (4), critical for implantation of the developing blastocyst (5–7). Progesterone levels peak around the 8-10th day following ovulation (8, 9). In a cycle without pregnancy, the corpus luteum subsequently decays and the falling steroid hormone levels trigger menstruation and the beginning of the next cycle. With implantation, rising levels of hCG maintain the corpus luteum, and progesterone levels remain high to provide optimal circumstances for the developing pregnancy. Between week 6 and 8 of gestation, the luteal-placental shift takes place (10, 11), and the pregnancy will no longer depend on the corpus luteum (12–14).

Hormone release from the developing follicles and the natural feedback mechanisms become essentially altered in artificially stimulated cycles and induced ovulation. Supraphysiologic serum estradiol levels during the ovarian stimulation phase may cause pituitary inhibition, thereby suppressing the natural pulsatile release of LH (15–17). The exogenous hCG trigger effectively mimics the action of the endogenous LH-peak and induces ovulation and formation of the corpus luteum. However, due to its longer circulating half-life, the exogenous hCG may cause sustained hyperstimulation of the corpus luteum, leading to early and supraphysiologic progesterone and estradiol levels and further hypothalamo-pituitary suppression in the early luteal phase (18). The corpus luteum is adequately supported by the exogenous hCG signal during the first half of the luteal phase, but once this signal is cleared approximately five to six days after injection, progesterone levels tend to drop considerably and early compared to the natural progesterone pattern (9). This may result in shortening of the luteal phase, and consequently, insufficient duration of exposure of the endometrium to progesterone. Given the crucial role of progesterone in creating optimal conditions for implantation, both the early peak and later presumed defective progesterone secretion may lead to premature arrival into the receptive state of the endometrium and insufficient maintenance of endometrium function. All this may potentially result in failure of early embryo implantation and growth (19).

These proposed mechanisms could be expected to have a more marked effect in gonadotropin-stimulated cycles than in cycles using CC for stimulation, since competitive binding of hypothalamic estrogen receptors by CC would mitigate the negative feedback exerted by elevated estradiol levels. Still, (mild) ovarian stimulation using gonadotropins has been shown to lead to higher live birth rates after IUI than stimulation with CC in couples with unexplained infertility (20). By treating the potential luteal phase defect in gonadotropin-stimulated cycles, the success rates of OS-IUI treatment might be further improved.

In IVF/ICSI treatment, luteal phase support (LPS) with exogenous progesterone positively affects progesterone serum levels and the length of the luteal phase, while low midluteal progesterone levels are associated with a low probability of live birth (21).

There is currently no consensus on the use of progesterone supplementation for luteal phase support in OS-IUI cycles. The most recent systematic review and meta-analysis on this subject was published in 2017 (22). The authors performed a rerun of their review from 2013 (23) and concluded that progesterone luteal phase support appeared to be beneficial in gonadotropin-stimulated, but not in CC-stimulated IUI cycles. Their analyses, however, did not include all eligible studies available to the authors at the time of search execution, pooled ongoing pregnancy outcome data from a large study (24) in syntheses of clinical pregnancy, disregarded ongoing pregnancy data from another study (25), did not count reported spontaneous pregnancies as events, assessed the statistical significance of the effect estimates for the different subgroups rather than testing formally for subgroup differences, did not address the implications of risk of bias for the confidence they placed on their conclusions, nor did they provide a formal analysis of the quality of the evidence. Several additional reports on LPS in OS-IUI were published in the past years, and the need for a definite verdict on the use of progesterone LPS is high. We therefore conducted the current systematic review and meta-analysis to collect additional evidence and applied revised methodology to firmly assess the effectivity and safety of progesterone luteal phase support in OS-IUI treatment for unexplained infertility and mild male factor.

## Methods

### Search strategy and selection criteria

This systematic review was conducted in accordance with the Preferred Reporting Items for Systematic Reviews and Meta-Analysis (PRISMA) statement (26) and prospectively registered with PROSPERO (CRD42021292325). A comprehensive search was performed in the bibliographic databases PubMed and Embase.com from inception to November 22nd, 2021. A re-run was conducted on the 8th of March, 2022. Citation searching was performed in Scopus. A search performed in CENTRAL and Cochrane Database of systematic reviews did not provide additional papers.

Search terms included controlled terms (MeSH in PubMed and Emtree in Embase) as well as free text terms. Search terms expressing ‘Progesterone’ and ‘Luteal phase support’ were used in combination with search terms comprising ‘Intrauterine insemination’ and ‘Ovarian stimulation’. The search was performed without date or language restrictions. A search filter was used to exclude animal studies. The full search strategies for all databases can be found in the Supplementary Information (**Supplementary A**).

### Study selection, critical appraisal, and data extraction

At least two reviewers (GC, TL, CL and KD) independently screened all potentially relevant titles and abstracts for eligibility using Rayyan (27). Studies were included if they were randomized controlled trials (RCTs), included couples undergoing OS-IUI treatment because of unexplained and/or mild male infertility, compared any form of progesterone luteal phase support to placebo or no intervention, and reported intention-to-treat, per-participant data on at least one of the included outcomes.

Studies were excluded if they only included females with cycle irregularities or PCO-syndrome or couples undergoing OS-IUI because of moderate male factor infertility, evaluated luteal phase support other than progesterone (e.g., hCG), compared different types or different dosages of LPS, without a placebo or no-intervention arm, evaluated other fertility treatments, such as IVF or ICSI, or did not allow for extraction of intention-to-treat, per participant outcome data, for instance because numbers of randomized participants per trial arm were missing. Conference abstracts without a published full-text article were excluded as well.

Risk of bias assessment for the primary outcomes was performed independently by at least two investigators (GC, TL, CL and KD), using the Cochrane ‘Risk of bias’ assessment tool 2.0 (28). Disagreements were resolved by discussion with a third reviewer. Risk of bias plots were created using robvis (29).

### Data synthesis and analysis plan

Data on trial design and setting, study population, participant baseline characteristics, OS-IUI treatment parameters, luteal phase interventions and dosages, and outcome data were extracted by at least two reviewers (GC, TL, CL and KD) working independently, using a previously designed and piloted data-extraction form. Primary outcomes were live birth and clinical pregnancy rates per randomized participant. In studies that did not report live birth, ongoing pregnancy was used as a proxy if reported. Secondary outcomes were multiple pregnancy rate, ectopic pregnancy rate, miscarriage rate, incidence of ovarian hyperstimulation syndrome (OHSS), side effects of progesterone luteal support, complications of pregnancy, perinatal outcomes, length of the luteal phase and mid-luteal progesterone serum levels.

Our primary analysis was conducted according to an intention-to-treat (ITT) principle. The number of randomized women per trial arm was used as the denominator for clinical pregnancy and live birth, and the subset of women with a clinical pregnancy in each trial arm for analyses of miscarriage, multiple pregnancy, and ectopic pregnancy. For studies that allowed multiple OS-IUI cycles, both first-cycle and cumulative outcome data were extracted if available. Cross-over designs were considered invalid and only first-cycle data were included in meta-analysis. Reported spontaneous pregnancies were counted as events in the analyses of cumulative outcomes, but not in single-cycle analyses.

We used Mantel-Haenszel random-effects models for meta-analysis of dichotomous outcomes and obtained the I^2^ statistic as a measure of between-study heterogeneity. Summary estimates of treatment effects were expressed as risk ratios (RR) with 95% confidence intervals (CI). Peto odds ratios (OR) with 95% CI were used for outcomes with rare events (< 1%) or zero cell counts. We used the Paule-Mandel estimator for the heterogeneity variance τ^2^ and applied Hartung-Knapp adjustments to obtain the 95% CI around the pooled effect estimate. 95% prediction intervals (PI) were obtained for all outcomes. Meta-regression was conducted with restricted maximum likelihood (REML) estimators and Hartung-Knapp adjustments. The results of meta-analyses were displayed as forest plots. Bubble plots were used for meta-regression. Results which could not be pooled were summarized in tables, figures, and text.

We planned in advance to perform the following analyses: I) An ITT meta-analysis of primary and secondary outcomes after a single OS-IUI cycle and cumulative over the total number of cycles reported in each study; II) An ITT subgroup analysis of primary outcomes to test for potential differences in the effectiveness of progesterone luteal phase support for the various ovarian stimulation agents; III) Meta-regression analysis to test for correlation at the study-level of effect size and daily dose of progesterone and duration of LPS treatment; and IV) Sensitivity analysis excluding studies at high risk of bias. Because it was impossible to extract data specifically for couples with unexplained infertility from most studies, we conducted a *post-hoc* subgroup analysis with studies that included up to 55% of participants with unexplained infertility, and studies in which the fraction of participants with unexplained infertility was above 95%. Motivated by the low statistical heterogeneity encountered in most analyses, we performed a *post-hoc* sensitivity analysis employing the DerSimonian-Laird estimator without Hartung-Knapp adjustment to control for potential type I errors (30).

Excel (version 2112) was used for data extraction and descriptive analysis. Meta-analyses were carried out in R version 4.0.3 (31) with packages “meta” (version 5.2-0) (32) and “dmetar” (version 0.0.9) (33). A quality of the evidence table including all prespecified outcomes was synthesized using the GRADEpro Guideline Development Tool (DGT) (34).

## Results

### Study and participant characteristics

**Figure 1:**
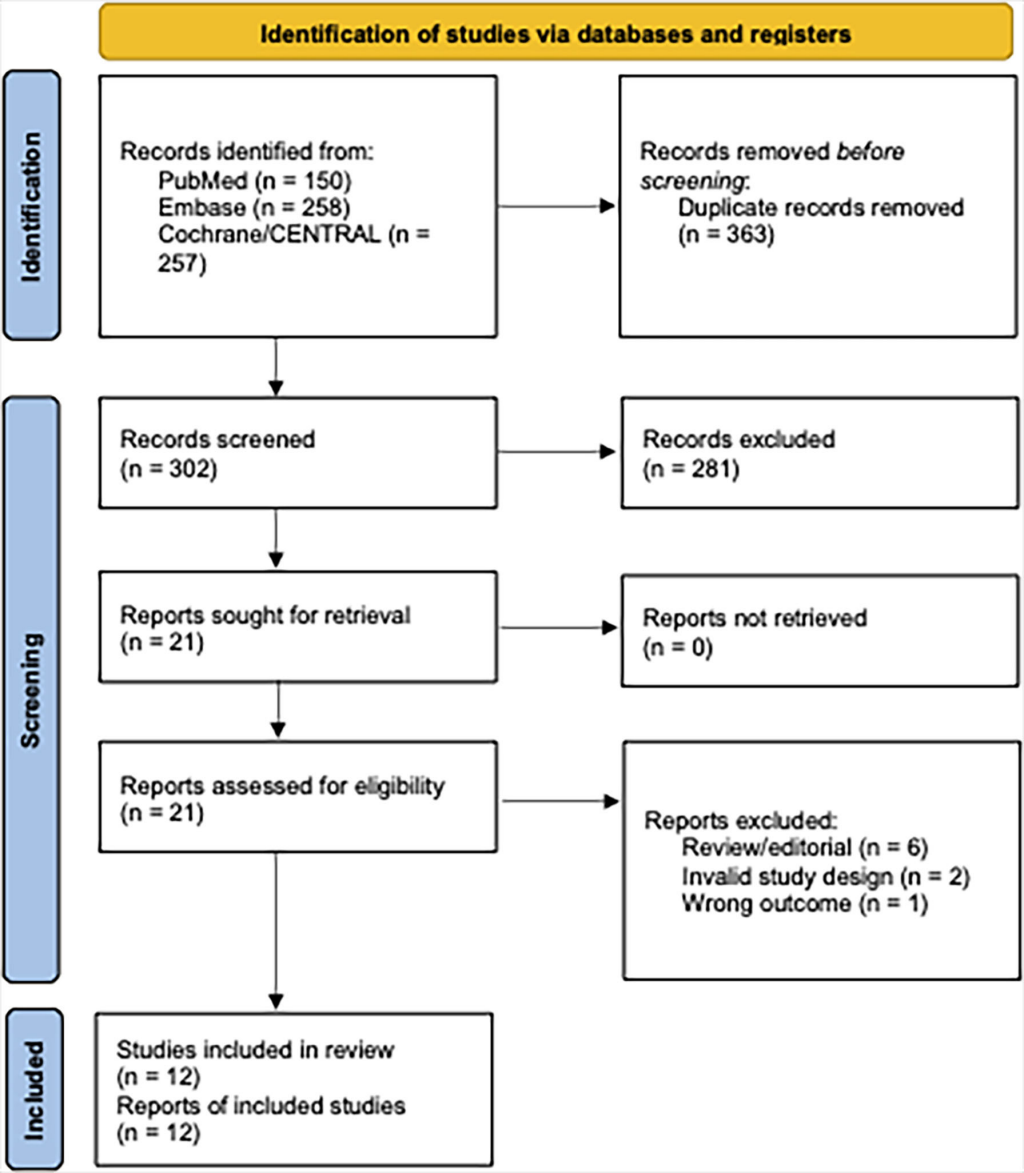
Flow diagram of identified and selected studies. Diagram adapted from (26).

Our systematic search yielded 665 records. After removal of duplicates, we screened 302 titles and abstracts and 21 full-text articles for eligibility. We identified fifteen published RCTs comparing progesterone luteal phase support after OS-IUI to placebo or no intervention which reported the outcomes of interest (9, 19, 24, 25, 35–45). With respect to the previous review by Green et al., we identified three additional studies (36, 43, 45) that met the inclusion criteria specified by the authors but were not included in their review, and one newer study (39).For the present review, we had specified unexplained or mild male infertility as the population of interest; this led to the exclusion of the study by Yacizi et al. (45), which included only women with PCOS. Our requirement that ITT, per participant data could be extracted resulted in exclusion of an additional two studies (36, 42). Ultimately, twelve studies met full inclusion criteria and were included in both qualitative and quantitative synthesis (**Figure 1**) (9, 19, 24, 25, 35, 37–41, 43, 44). Design features and demographic characteristics of the included studies are summarized in **Table 1**. An overview of baseline participant characteristics and treatment parameters is given in **Supplementary Table I**.

**Table 1:** Characteristics of studies included in meta-analyses.

Study ID	Country	Single/Multi center	Recruitment period	Study population	No. of cycles allowed	Ovulation induction	hCG trigger dose (IU)	Type of progesterone	Dose (mg/day)	End of treatment	Furthest stage of pregnancy reported	No. of patients	No. of completed cycles
Aali 2013	Iran	S	Apr 2010 – Dec 2011	Undergoing IUI, less than two previous failed cycles	1	hMG or CC	10.000	Cyclogest (Pessaries)	400	Day 10	CP	196	196
Agha-Hosseini 2012	Iran	M	Apr 2009 – Nov 2010	Unexplained infertility or mild male factor	1	CC/letrozole/CC + hMG/letrozole + hMG	10.000	Cyclogest (pessaries)	400	Week 12	CP	300	290
Ebrahimi 2010	Iran	S	Oct 2007 – Dec 2008	Unexplained infertility	3	CC + hMG	5.000	Cyclogest (pessaries)	400	Week 10	LB	200	511
Erdem 2009	Turkey	S	Nov 2004 – Oct 2006	Unexplained infertility	3	rFSH (Gonal-f)	10.000	Crinone (vaginal gel)	90	Week 12	LB	214	427
Karadag 2016	Turkey	S	No information	Unexplained infertility	1	CC or rFSH (Gonal-f)	6.500	Crinone (vaginal gel)	90	Week 10	LB	200	200
Keskin 2020	Turkey	S	Aug 2014 – Jan 2015	Unexplained infertility or male subfertility, undergoing first IUI cycle	1	hMG/rFSH (Gonal-f or Puregon)/hpHMG	10.000	Progestan (vaginal capsules)	200	Confirmed vital pregnancy	CP	87	87
Kyrou 2010	Belgium	S	Sep 2008 -Dec 2009	Normo-ovulatory, undergoing first IUI with CC	1	CC	5.000	Utrogestan (vaginal capsules)	600	Week 7	OP	468	400
Maher 2012	Saudi Arabia	S	Jun 2008 – Mar 2010	Undergoing first IUI cycle	6^1^	rFSH (Gonal-f)	10.000	Crinone (vaginal gel)	90	Day 14	LB	71	258
Peeraer 2016	Belgium	M	Apr 2011 – Jan 2015	Normo-ovulatory, undergoing first IUI cycle	1	rFSH (Gonal-f)	6.500	Crinone (vaginal gel)	90	Day 15	LB	393	364
Rashidi 2014	Iran	S	Jan 2012 – Dec 2012	Undergoing IUI	1	CC + hMG	10.000	Vaginal progesterone	800	Week 8	OP	253	253
Schwarze 2013	Chile	M	No information	Unexplained infertility, undergoing first IUI cycle	1	rFSH (Puregon)	5.000	Fertiring (vaginal ring)	10	Week 12	CP	100	100
Seckin 2014	Turkey	S	Sep 2010 – Jun 2011	Unexplained infertility	3	rFSH (Gonal-f)	10.000	Crinone (vaginal gel)	90	Week 12	LB	149	166

1 Cross-over design: first cycle randomized, thereafter alternating.

2 Comparator: placebo. All other studies compared progesterone LPS to no intervention.

S, single center; M, multicenter; IUI, intrauterine insemination; NoCC, clopmiphene citrate; hMG, human menopausal gonadotropin; hpHMG, highly purified hMG; rFSH, recombinant follicle stimulating hormone; hCG, human chorionic gonadotropin; IU, international unit; CP, clinical pregnancy; OP, ongoing pregnancy; LB, live birth.

The included studies were 9 single-centre and 3 multicentre RCTs from Iran, Turkey, Belgium, Saudi Arabia, and Chile. Eight trials studied a single cycle of OS-IUI. One trial (40) allowed participants to attempt up to 6 OS-IUI cycles; participants were randomly allocated to the intervention or control groups for the first cycle and alternated between trial arms in each consecutive cycle thereafter. Only first-cycle data were extracted from this study, assessed for risk of bias, and included in meta-analyses of both single-cycle and cumulative outcomes. The remaining three studies (19, 37, 44) allowed up to 3 cycles per participant and did not permit participants to switch between trial arms. The studied populations were heterogeneous, with different proportions (21-100%) of couples with unexplained infertility. None of the studies presented outcome data separately for patients with different infertility diagnoses; hence, data for couples with unexplained infertility and mild male factor could not specifically be extracted as intended. Sample sizes varied between 71 - 468 participants (median: 200). Only one trial (25) was placebo-controlled. Six studies (37, 39–41, 43, 44), used gonadotropins (i.e. FSH or human menopausal gonadotropin (hMG)) for stimulation; one study (38) used either gonadotropins or CC; one study (24) used CC, and three studies (9, 19, 25), used a combination of CC and gonadotropins; the remaining study (35) consisted of four groups that used CC, letrozole, CC + hMG, or letrozole + hMG for stimulation. The dose of hCG employed to trigger ovulation varied between 5.000 – 10.000 IE (median: 10.000). All twelve studies used vaginal progesterone for luteal phase support, but the administration form (pessary, capsules, gel, ring), dosage (10 – 800 mg/day, median: 145), and duration of treatment (10 days – 3 months, median: 6 weeks) differed greatly among studies.

### Quality assessment and risk of bias from included studies

Of the twelve studies included in meta-analysis, two trials were prospectively registered (25, 41) and four were retrospectively registered (9, 19, 24, 35), the remaining six trials did not provide any information on registration. All twelve trials reported obtaining IRB approval. There was only one double-blind trial (25), and a second trial (9) reported blinding of the researcher.

**Figure 2:**
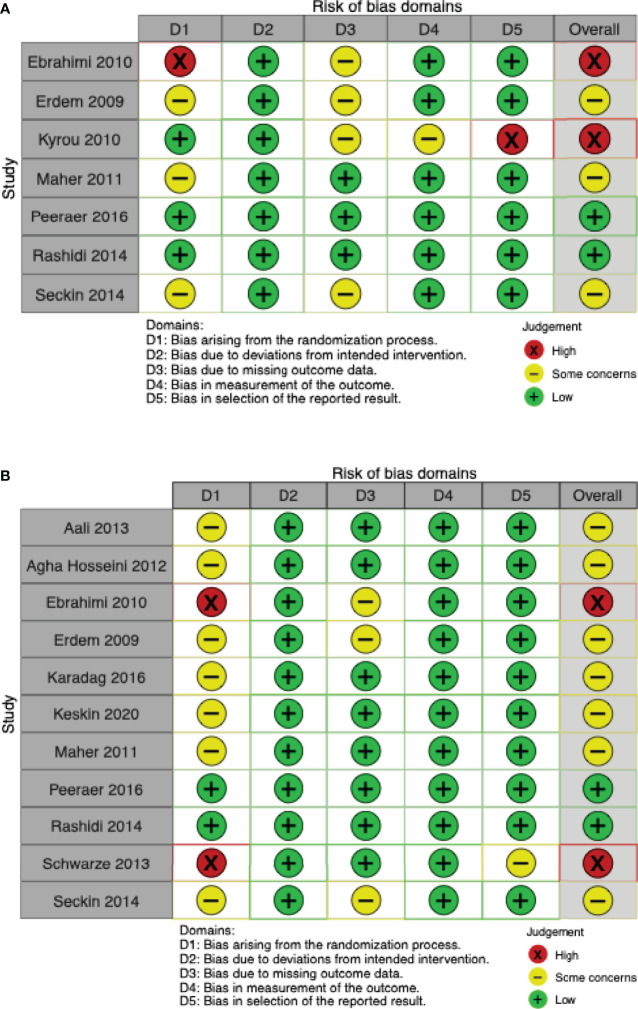
Risk of bias ‘traffic-lights’ plots for the studies included in meta-analysis. **(A)** Live birth. **(B)** Clinical pregnancy.

Five studies (19, 37, 40, 41, 44) reported live birth rates, and two additional studies (24, 25) reported ongoing pregnancy rates. Live birth data from one trial (19) were deemed to be at high risk of bias because of the described sequential randomization. Ongoing pregnancy data from the study by Kyrou et al. (24) were deemed to be at high risk of bias because of premature termination of the trial after an interim analysis. Three other studies raised some concerns because of unclear or lacking information on allocation sequence concealment (37, 40, 44), or because of missing outcome data (37, 44). Live birth data from the study by Peeraer et al. (41) were deemed to be at low risk of bias (**Figure 2A**).

Eleven of twelve studies included in this meta-analysis reported clinical pregnancy rates (9, 19, 24, 25, 35, 37–41, 43, 44). Two trials (25, 41), were at overall low risk of bias. Three studies were found to be at high risk of bias: one (19) used sequential randomization; one (43) did not provide any details on the randomization process and the baseline table showed differences (younger age, younger partner age, shorter duration of infertility) that favoured the intervention group. Due to unclear or missing details on allocation sequence concealment or missing outcome data, the remaining seven studies were judged as “some concerns” (9, 25, 37–40, 44) (**Figure 2B**).

### Risk of bias from missing data

Potential sources of bias from missing data in our results are: 1) our decision to exclude studies with no usable data (36, 42) (; 2) missing outcome data (e.g., single-cycle data from Ebrahimi et al. and Seckin et al. (19, 44), clinical pregnancy data from Kyrou et al. (24)); 3) missing baseline data (e.g. Maher); and 4) missing studies, though our search was comprehensive and the funnel plots we constructed did not present any obvious asymmetries and thus did not suggest the presence of publication bias or other small-study effects (**Supplementary Figures 1A, 1B**). However, due to the small number of studies, the use of a funnel plot to assess risk of bias due to non-reporting may not be appropriate, especially for the outcome live birth.

### Results of meta-analysis

#### Main outcomes: live birth and clinical pregnancy rates

**Figure 3:**
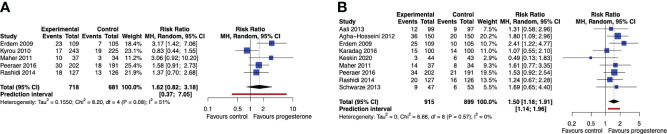
Live birth and clinical pregnancy rate after a single OS-IUI cycle. Comparison: progesterone luteal phase support versus placebo or no intervention. **(A)** Forest plot of live birth. **(B)** Forest plot of clinical pregnancy. MH, Mantel-Haenszel; CI, confidence interval.

We found insufficient statistical evidence that progesterone luteal support after IUI, compared to no luteal support or placebo, improves the chance of a live birth in a single cycle of OS-IUI (RR 1.62, 95% CI [0.82, 3.18], 95% PI [0.37, 7.05]; I^2^ = 51%; 5 RCTs, 1399 participants; very low quality evidence) (**Figure 3A**), although it improved the chance of a clinical pregnancy (RR 1.50, 95% CI [1.18, 1.91], 95% PI [1.14, 1.96]; I^2^ = 0%; 9 RCTs, 1814 participants; moderate quality evidence) (**Figure 3B**). These results suggest that for a live birth rate of 9% per OS-IUI cycle without luteal support, the average live birth rate with progesterone luteal support would become between 7% and 29%. Likewise, for a clinical pregnancy rate of 12% per OS-IUI cycle without luteal support, an average clinical pregnancy rate between 14% and 23% would be achieved with application of progesterone luteal phase support.

**Figure 4:**
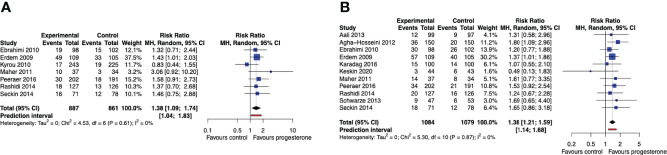
Cumulative live birth and clinical pregnancy rate. Comparison: progesterone luteal phase support versus placebo or no intervention. **(A)** Forest plot of live birth. **(B)** Forest plot of clinical pregnancy. MH, Mantel-Haenszel; CI, confidence interval.

When data across the entire study period were pooled regardless of the number of OS-IUI cycles, cumulative live birth rates for up to 3 OS-IUI cycles were improved for women treated with progesterone luteal support (RR 1.38, 95% CI [1.09, 1.74], 95% PI [1.04, 1.83]; I^2^ = 0%; 7 RCTs, 1748 participants; moderate quality evidence) (**Figure 4A**). Progesterone luteal support similarly improved the cumulative chance of a clinical pregnancy (RR 1.38, 95% CI [1.21, 1.59], 95% PI [1.14, 1.68]; I^2^ = 0%; 11 RCTs, 2163 participants; moderate quality evidence) (**Figure 4B**). These results imply that for a cumulative live birth rate of 10% after a series of OS-IUI cycles without luteal support, the average cumulative live birth rate with progesterone luteal support would become between 11% and 17%. Likewise, for a cumulative clinical pregnancy rate of 14% without luteal support, an average clinical pregnancy rate between 17% and 22% would be achieved with application of progesterone luteal phase support.

#### Secondary outcomes

Only cumulative outcome data for our secondary outcomes were available from multi-cycle trials. We found no evidence of an effect of progesterone luteal phase support on multiple pregnancy rate (Peto OR 0.78, 95% CI [0.38, 1.59], 95% PI [0.25, 2.40]; I^2^ = 0%; 8 RCTs, 374 clinical pregnancies; very low-quality evidence) or on miscarriage rate (Peto OR 0.89, 95% CI [0.56, 1.41], 95% PI [0.45, 1.75]; I^2^ = 0%; 9 RCTs, 410 clinical pregnancies; low-quality evidence) (**Supplementary Figures 2** and **3**). Three studies (35, 40, 44) reported the incidence of ectopic pregnancies. Only 3 ectopic pregnancies were reported in total, all in the study by Maher et al. (40), all three in the control group; therefore, meta-analysis of ectopic pregnancy rate could not be undertaken. Four studies explicitly reported that none of the participants developed OHSS ( (19, 37, 40, 44). None of the studies reported side effects of progesterone, nor perinatal or pregnancy outcomes.

Only one of the included articles in this systematic review (9) reported serum progesterone levels. Serum progesterone on day 7 after hCG injection was significantly higher in the group receiving LPS (48.34 versus 34.49 nmol/L respectively, p<0.001). This difference persisted until day 11 after the hCG trigger. Two studies reported luteal phase duration as an outcome (9, 41). The luteal phase was significantly longer in LPS cycles compared to non-supported cycles (Aali: 14.46 ± 1.7 days versus 13.05 ± 1.4 days, p<0.037, Peeraer: 16.6 ± 2.2 versus 14.6 ± 2.5 days, p<0.001).

#### Subgroup analysis: stimulation drug

**Figure 5:**
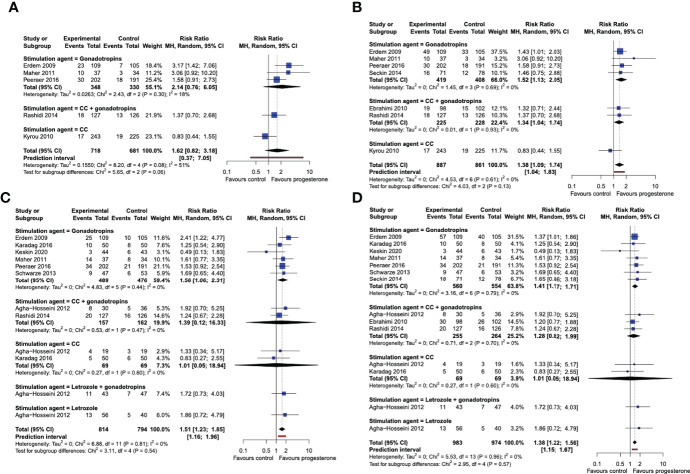
Subgroup analysis by stimulation drug. Comparison: progesterone luteal phase support versus placebo or no intervention. **(A)** Forest plot of live birth, single/first cycle. **(B)** Forest plot of live birth, cumulative over all study cycles. **(C)** Forest plot of clinical pregnancy, single/first cycle. **(D)** Forest plot of clinical pregnancy, cumulative over all study cycles. Subgroups by stimulation agent. CC, clomiphene citrate; MH, Mantel-Haenszel; CI, confidence interval.

When the studies were analyzed in subgroups according to the medication used for ovarian stimulation, we did not find sufficient statistical evidence that live birth rates after a single OS-IUI cycle were improved by progesterone luteal phase support in any of the subgroups (**Figure 5A**). The cumulative live birth rate was improved by progesterone luteal phase support in the subgroup of patients stimulated with gonadotropins (RR 1.52, 95% CI [1.13, 2.05]; I^2^ = 0%; 4 RCTs, 827 participants; moderate-quality evidence), and in the subgroup stimulated with CC + gonadotropins (RR 1.34, 95% CI [1.04, 1.74]; 2 RCTs, 453 participants; very low-quality evidence), but not in the subgroup stimulated with CC (RR 0.83, 95% CI [0.44, 1.55]; 1 RCT, 468 participants; very low quality evidence) (**Figure 5B**). The Chi-square test for subgroup differences was not significant, meaning that there is insufficient statistical evidence of a differential effect of progesterone on live birth rates for the various ovarian stimulation protocols. However, it should be noted that due to the low number and uneven distribution of studies that contributed data to the studied subgroups, this analysis could not be expected to detect a potential effect difference.

Progesterone luteal phase support improved the chance of a clinical pregnancy after a single OS-IUI cycle in the gonadotropins subgroup (RR 1.56, 95% CI [1.06, 2.31]; I^2^ = 0%; 6 RCTs, 965 participants; moderate-quality evidence), but not in the gonadotropins + CC (RR 1.39, 95% CI [0.12, 16.33]; I^2^ = 0%; 2 RCTs, 319 participants; low-quality evidence), nor in the CC only (RR 1.01, 95% CI [0.05, 18.94]; I^2^ = 0%; 2 RCTs, 138 participants; low-quality evidence) subgroups. (**Figure 5C**) The Chi-square test for subgroup differences was not significant (P = 0.81). The cumulative clinical pregnancy rate was higher in progesterone luteal phase supported cycles in the gonadotropin-stimulated subgroup (RR 1.41, 95% CI [1.17, 1.71]; I^2^ = 0%; 7 RCTs, 1014 participants; moderate-quality evidence), but not in the gonadotropins + CC (RR 1.28, 95% CI [0.82, 1.99]; I^2^ = 0%; 3 RCTs, 519 participants; low-quality evidence), nor in the CC only subgroup (RR 1.01, 95% CI [0.05, 18.94]; I^2^ = 0%; 2 RCTs, 138 participants; low-quality evidence). The Chi-square test for subgroup differences was again not significant (P = 0.59) (**Figure 5D**). Thus, our analysis does not provide statistical evidence that the stimulation drug used in OS-IUI modifies the effect of progesterone luteal phase support on clinical pregnancy rate. Because a considerably larger number of trials and participants contributed data to the gonadotropin-stimulated subgroup than to any other subgroup, and only one study contributed data to the letrozole and letrozole + gonadotropins-stimulated subgroups, our analysis may lack the power to detect potential differences between these stimulation subgroups.

#### Meta-regression analysis: progesterone dosage and duration of treatment versus effect size

To test the hypothesis that differences in the estimated effect sizes between the studies may be partially due to differences in the employed dosage, we plotted effect size against administered daily dose of progesterone for the studies included in syntheses of our main outcomes (**Supplementary Figures 4A, B**). Visual examination of the plots revealed no apparent relationship, linear or otherwise, between the daily dose of progesterone employed in a study and the observed effect size. In meta-regression mixed-effects models fitted to cumulative live birth and clinical pregnancy outcomes, progesterone dose did not predict the observed effect size (regression weight in model for live birth: -0.0005, 95% CI [-0.0014, 0.0004], p = 0.2069; for clinical pregnancy rate: -0.0001, 95% CI [-0.0008, 0.0006], p = 0.6779). Similarly, the duration of progesterone LPS between the included studies did not explain the differences in effect size (regression weight in model for live birth: -0.0059, 95% CI [-0.0926, 0.0809], p = 0.8688; for clinical pregnancy rate: 0.0084, 95% CI [-0.0370, 0.0538], p = 0.6838) (**Supplementary Figures 4C, D**).

#### *Post-hoc* subgroup analysis: fraction of unexplained infertility

Six studies included participants with several underlying causes of infertility or other indications for IUI treatment, and did not report outcomes separately for each diagnosis (9, 24, 25, 35, 40, 41). The proportion of couples with unexplained infertility in these studies varied between 21% and 55%. The RR for cumulative live birth rate comparing progesterone LPS with no intervention or placebo in this subgroup of studies was 1.36 (95% CI 0.66 to 2.79, I^2^ = 32%). The remaining six studies included almost exclusively (> 95%) couples with unexplained infertility (19, 37–39, 43, 44). In this second subgroup, the RR for cumulative live birth rate was 1.41 (95% CI 1.27 to 1.58, I^2^ = 0). We observed no statistically significant subgroup effect (p = 0.87) (**Supplementary Figure 5A**). Likewise, the RR for clinical pregnancy rate in studies with up to 55% of couples with unexplained infertility was 1.52 (95% CI 1.26 to 1.85, I^2^ = 0). In studies with a high proportion of unexplained infertility, the RR for clinical pregnancy rate when comparing progesterone LPS with no intervention or placebo was 1.30 (95% CI 1.03 to 1.64, I^2^ = 0). There was no statistically significant difference between the subgroups (p = 0.63) (**Supplementary Figure 5B**).

#### Sensitivity analyses

We assessed whether our results would change by including only studies with a moderate to low risk of bias. Excluding studies deemed to be at high risk of bias (19, 24, 43) from our syntheses yielded comparable effect estimates that were the same, or larger than those obtained from the analysis of all included studies. In order to test whether the results of our meta-analysis did not depend on our model choice, we performed a sensitivity analysis using DerSimonian-Laird estimators without Hartung-Knapp adjustments in random effects models for the syntheses of the main outcomes. Significance was only affected in the meta-analysis of live birth rate after a single OS-IUI cycle, for which the DerSimonian-Laird method yielded a significant effect estimate (RR 1.61, 95% CI [1.01, 2.56], 95% PI [0.39, 6.58]), whereas the Paule-Mandel method with Hartung-Knapp adjustment did not (RR 1.62, 95% CI [0.82, 3.18], 95% PI [0.37, 7.05]. For the remaining three outcomes, both models yielded very similar effect estimates and did not affect significance (**Table 2**).

**Table 2:** Sensitivity analyses.

Outcome	RR [95% CI]PM + hakn	RR [95% CI]DL	RR [95% CI]PM + haknExcluding high RoB^1^
Live birth rate, single cycle	1.62 [0.82, 3.18]	1.61 [1.01, 2.56]	1.88 [0.99, 3.58]
Clinical pregnancy rate, single cycle	1.50 [1.18, 1.91]	1.50 [1.20, 1.87]	1.49 [1.14, 1.95]
Live birth rate, cumulative	1.38 [1.09, 1.74]	1.38 [1.11, 1.71]	1.50 [1.21, 1.86]
Clinical pregnancy rate, cumulative	1.38 [1.21, 1.59]	1.38 [1.17, 1.64]	1.41 [1.19, 1.66]

1 Excluded studies (19, 24, 43):

RR, risk ratio; CI, confidence interval; PM, Paule-Mandel estimator for τ^2^; hakn, Hartung-Knapp correction; DL, DerSimonian-Laird estimator for τ^2^; RoB, risk of bias.

## Discussion

### Principal findings

The findings of this systematic review and meta-analysis suggest that progesterone luteal phase support after OS-IUI applied in a single cycle or in a series of consecutive cycles leads to higher live birth and clinical pregnancy rates than no luteal support or placebo. This effect is specifically present in protocols using gonadotropins for ovarian stimulation. Since most of the data on CC-stimulated cycles came from a single study deemed to be at high risk of bias (24) we consider that the evidence of an effect of progesterone luteal phase support in protocols involving CC is inconclusive, and that stating that such an effect is unlikely to exist, as has been previously suggested (22), is premature. The preferred dosage and optimal duration of progesterone treatment remain unknown based on the current data. Statistical heterogeneity was low for most outcomes, suggesting that our estimates may approximate a common true effect of progesterone LPS. This effect may prove consistent across different populations and variations in treatment parameters. Our results also proved robust under the different models and assumptions tested in the sensitivity analysis. However, the low level of statistical heterogeneity in our meta-analysis cannot and should not be seen as additional proof of a potential effect of progesterone LPS. There is considerable clinical and methodological between-study heterogeneity. In such situations, low statistical heterogeneity has been shown to not be predictive of the reliability of the results (46). The overall moderate study quality and small to moderate size of the individual studies demand caution in weighing the certainty of the evidence, and imply that broad implementation of progesterone luteal support in OS-IUI treatment may not be justified with the current research data. A large, well-designed, well-powered trial could provide the decisive evidence that is still needed to either raise the quality to “high” and confirm the effect of progesterone LPS in OS-IUI, or dismiss the apparent effect as one more sad example of irreproducible research.

We did not find evidence of an effect of progesterone luteal support on multiple pregnancy or miscarriage rates in OS-IUI cycles, but the evidence for these outcomes is of low to very low quality, and an effect in either direction cannot be fully ruled out. Data on ectopic pregnancy rates, OHSS, serum progesterone levels, length of luteal phase, side effects and (pregnancy) complications are too sparse to review. An overview of the results can be found in **Table 3**.

**Table 3:** Summary of evidence.

Outcome	Certainty assessment							№ of patients		Effect		Certainty
	№ of studies	Study design	Risk of bias	Inconsistency	Indirectness	Imprecision	Other considerations	Main analysis: ITT, progesterone	control	Relative(95% CI)	Absolute(95% CI)	
Live birth, single cycle	5	randomised trials	very serious^a^ ^,^ ^b^	serious^c^	not serious	not serious	none	98/718 (13.6%)	60/681 (8.8%)	**RR 1.62** (0.82 to 3.18)	**55 more per 1,000** (from 16 fewer to 192 more)	⨁◯◯◯ Very low
Clinical pregnancy, single cycle	9	randomised trials	serious^a^ ^,^ ^d^	not serious	not serious	not serious	none	168/915 (18.4%)	110/899 (12.2%)	**RR 1.50** (1.18 to 1.91)	**61 more per 1,000** (from 22 more to 111 more)	⨁⨁⨁◯ Moderate
Live birth, cumulative	7	randomised trials	serious^a^ ^,^ ^d^	not serious	not serious	not serious	none	159/887 (17.9%)	113/861 (13.1%)	**RR 1.38** (1.09 to 1.74)	**50 more per 1,000** (from 12 more to 97 more)	⨁⨁⨁◯ Moderate
Clinical pregnancy, cumulative	11	randomised trials	serious^a^ ^,^ ^d^	not serious	not serious	not serious	none	248/1084 (22.9%)	178/1079 (16.5%)	**RR 1.38** (1.21 to 1.59)	**63 more per 1,000** (from 35 more to 97 more)	⨁⨁⨁◯ Moderate
Multiple pregnancy, cumulative	8	randomised trials	serious^a^, ^d^	not serious	not serious	very serious^e^	none	14/216 (6.5%)	13/158 (8.2%)	**OR 0.78** (0.35 to 1.72)	**17 fewer per 1,000** (from 52 fewer to 51 more)	⨁◯◯◯ Very low
Miscarriage, cumulative	9	randomised trials	serious^a^ ^,^ ^d^	not serious	not serious	serious^f^	none	37/236 (15.7%)	30/174 (17.2%)	**OR 0.89** (0.56 to 1.41)	**16 fewer per 1,000** (from 68 fewer to 55 more)	⨁⨁◯◯ Low

CI, confidence interval; OR, Peto odds ratio; RR, risk ratio.

Explanations

a Most studies raise some concerns.

b Likely at risk of bias from non-reporting.

c There was substantial statistical heterogeneity.

d Some studies are at high risk of bias.

e Very small total number of events.

f Small total number of events.

### Serum progesterone and pregnancy

The true pathophysiology of luteal phase deficiency and the optimal serum progesterone concentration to achieve and maintain a successful pregnancy remain largely unknown (47). Luteal phase serum progesterone fluctuates throughout the day and there is a wide intra- and interindividual variation (48, 49). Low progesterone levels in the mid-luteal phase have been linked to substandard live birth rates after OS-IUI (21, 50), but it is unclear whether a low progesterone concentration is the consequence or cause of non-implantation. Due to the scarcity of data on serum progesterone levels in the included studies, these questions could not be adequately addressed in the present meta-analysis. The only included study that reported serum progesterone levels (9) found a mean serum progesterone concentration in the control group on day 11 after hCG injection (21 nmol/L, versus 34 nmol/L in the LPS group) that was lower than the critical threshold of 25 nmol/L determined by Costello et al. (50); however, the difference in clinical pregnancy rates between the groups did not reach statistical significance, likely due to the low study power.

Progesterone is also important to preserve early pregnancy, and its premature withdrawal may be associable to miscarriage. In this meta-analysis, we found no significant effect of progesterone LPS on miscarriage rates. This is consistent with the findings from the PRISM trial (51) and a recent network meta-analysis (52) by the same authors that progesterone may not be useful in preventing miscarriage or early pregnancy loss in the general population. For women with a history of one or more previous miscarriages and early pregnancy bleeding, however, vaginal micronized progesterone resulted in higher live birth rates than placebo. Even in a large-scale trial like the PRISM (4153 participants), this effect became apparent only after deliberate subgroup analysis. It is therefore not strange that we have not observed such an effect.

### Comparison with previous reviews and other studies

Live birth rates and clinical pregnancy rates may improve when LPS is applied. Several reviews previously evaluated the efficacy of progesterone LPS in IUI with stimulated cycles (22, 23, 53) and, in general, established comparable results. Two individual studies demonstrated a statistically significant effect of LPS on clinical pregnancy and/or live birth rates (35, 37), while most other studies reported improved clinical pregnancy and/or live birth rates that were not statistically significant at the 0.05 significance level. No population parameters or design features were identified that satisfactorily explained this difference in significance. Erdem et al. (37) included couples with a relatively long duration of infertility (on average 4.7-5.0 years), and it has been suggested previously that couples dealing with a long period of infertility might be at increased risk of luteal phase deficiency, and thus may specifically benefit from LPS. Nevertheless, several other studies included a relatively long mean duration of infertility (e.g. 4.8 years (19), 4.6 years (44)) or included patients with a minimum of two years of infertility (39, 40) and failed to report a significant result independently.

The percentage of patients with unexplained infertility differed greatly across the included studies. This may have caused bias, as the preferred ovarian stimulation method and overall chance to achieve pregnancy differ across patient groups. In our subgroup analysis, progesterone LPS improved live birth rate and clinical pregnancy rate to a similar extent in studies with a sample consisting almost exclusively of couples with unexplained infertility, and in studies that included a diversity of infertility diagnoses, suggesting that the putative beneficial effect of LPS, if confirmed by future studies, may be generalizable to several of the patient groups for whom OS-IUI is the indicated (first-line) treatment.

Generally, CC is the preferred stimulation agent for anovulatory or oligo-ovulatory patients, whilst gonadotropins are usually applied in OS-IUI for unexplained infertility. As proposed previously (22), there might be a difference in the benefit of progesterone LPS following gonadotropin-stimulated or CC-stimulated cycles, though evidence at this time is inconclusive. As exposed in the introduction, such a difference would be consistent with the known mechanisms of action of these preparations (54). Furthermore, the proposed negative feedback resulting from supraphysiologic estradiol levels may be more pronounced in stimulated cycles with multifollicular development, as multiple corpora lutea cause higher luteal phase estradiol levels. Seckin et al. (44) only found a statistically significant effect of LPS on clinical pregnancy if the analyses were stratified according to the number of developing dominant follicles (28.2% in multifollicular cycles vs 11.4% in monofollicular cycles, p=0.04). Nonetheless, Bakay et al. (36) specifically focused their research on mono-follicular cycles and also reported benefits of LPS in OS-IUI, although serious concerns regarding the quality of this study should be mentioned. Meanwhile, the study performed by Peeraer et al. (41), deemed to be at low risk of bias, found comparable results in cycles with monofollicular and multifollicular response (live birth rate RR 1.75; 95% CI, 0.84–3.62; P=0.133 in treatment group versus control group, monofollicular response cycles and live birth rate RR 1.09; 95% CI, 0.32–3.75; P=0.892 in multifollicular response cycles, respectively). In the present meta-analysis, data on dominant follicle number were insufficient to perform an additional subgroup analysis. More studies are needed to further assess this theory.

At present, there is no consensus on the optimum dose, administration route and form, or duration of treatment for luteal support. Several administration routes are available, such as vaginal, oral, intramuscular, or subcutaneous (55). Based on the most recent Cochrane Review, there is insufficient evidence to recommend any one route of administration over the other (56). All the included studies used (various forms of) vaginal progesterone. In principle, different forms of administration, variable formulation, and the wide range of progesterone dosages used, could have affected the findings of the studies included in this review. However, based on our meta-regression analysis, there was no apparent study-level correlation between progesterone dosage and the observed effect size.

We found no relationship either between duration of treatment and the observed effect size. The onset of treatment ranged from day of IUI to IUI+2 days, and end of treatment ranged from IUI+10 days to IUI+12 weeks. Preferred start- and duration of LPS have not been adequately studied and remain a matter of debate. In stimulated cycles, corpus luteum insufficiency and defective progesterone production may particularly arise during the first days and weeks of the luteal phase. LPS may not be of added value after the luteal-placental shift, and progesterone treatment may be safely ended at 6-8 weeks of gestation or even earlier. A recent meta-analysis of seven trials involving 1627 participants (57) found similar clinical outcomes between early progesterone cessation (week 4-7 of gestation) and progesterone continuation (up to week 12 of gestation) in fresh embryo transfers after IVF/ICSI treatment (live birth rate: RR 0.94, 95% CI 0.88–1.00; miscarriage rate: RR 0.91, 95% CI 0.69–1.20; ongoing pregnancy rate: RR 0.98, 95% CI 0.91–1.05) (57). Nevertheless, results were limited by sample size and may not be applicable to other forms of medically assisted reproduction.

There were no data available on side-effects, complications of pregnancy and perinatal outcome following OS-IUI with LPS. None of the studies included in this meta-analysis reported a higher multiple gestation rate in the progesterone-supported cohorts. Other trials that evaluated the use of vaginal progesterone capsules during early pregnancy (e.g. the PRISM and PROMISE trial), did not demonstrate any significant increase in risk of pregnancy or adverse neonatal outcomes (58, 59). Adverse effects of LPS in other reproductive treatments (e.g. IVF/ICSI cycles) are also poorly reported (56). Nonetheless, LPS appears to be safe according to long-term offspring health studies following IVF/ICSI treatment (where LPS with natural progesterone has been widely applied), as there is no presumed effect of progesterone LPS on health outcomes (e.g.: birth weight, preterm birth below 37 or 32 weeks of gestation, congenital malformations, perinatal mortality) (60, 61).

### Strengths and limitations

We have conducted a comprehensive and updated systematic review and meta-analysis of published RCTs reporting on live birth and clinical pregnancy rates after progesterone luteal phase support in OS-IUI treatment. Used methods are in line with the PRISMA statement (26) and the entire review process was executed by at least two independent reviewers. With respect to the previous systematic review by Green et al. (22), our analysis contains several methodological improvements: 1) Live birth and clinical pregnancy rates per patient were extracted and synthesized both after a single OS-IUI cycle (more comparable outcome measures) and after a series of cycles (more clinically relevant). 2) We performed all analyses according to a strict intention-to-treat principle and rejected those studies from which intention-to-treat, per-participant data could not be extracted. 3) Ongoing pregnancy outcome data were pooled with live birth (as opposed to clinical pregnancy) data, for which they have been shown to be a better proxy (62–64). 4) We used the Paule-Mandel estimator for the between-study variance and applied Hartung-Knapp adjustments to calculate 95% confidence intervals, which follows current recommendations (65). 5) We assessed risk of bias from the included studies on a per-outcome basis using the updated Cochrane RoB 2.0 tool and considered several possible sources of non-reporting bias.

The main limitation of this meta-analysis stems from the overall quality of the evidence. Using GRADE, we assessed the evidence for most of our outcomes to be of low to moderate quality due to risk of bias and imprecision (**Table 3**). Additionally, there was insufficient power to perform adequate subgroup analyses, so that it remains unknown which administration route, dosage, and treatment duration should be favoured for LPS in OS-IUI.

### Clinical implications and implications for research

The results of the present review and meta-analysis suggest benefit of progesterone LPS in OS-IUI treatment. Both clinical pregnancy and live birth rates were improved, and the risks of a multiple pregnancy or of a miscarriage were not affected. Based on our findings and considering the favorable safety profile, universal availability, and low cost of progesterone preparations, it may seem feasible to recommend the use of luteal support in all OS-IUI cycles. However, progesterone luteal phase support also brings a certain level of burden of treatment to the patient, comprising side-effects like vaginal discharge, fatigue, and headaches. Since the desired level of evidence quality has not yet been attained, additional trials with sufficient power are mandatory to confirm the findings in this meta-analysis and justify implementation of progesterone LPS in daily practice. We recommend including multiple OS-IUI cycles to report per-patient outcomes that are clinically relevant. The use of a cross-over design should be avoided as success (defined as a successful pregnancy) in the first phase of the trial precludes participation in the second phase, which causes unreliable results. On July 18^th^, 2022, the authors have obtained a grant from ZonMW to execute a trial as just described (registered as NCT05080569, NL9766)

### Conclusions

Low to moderate quality of evidence from this meta-analysis suggests that progesterone luteal phase support may be effective and safe in OS-IUI, leading to increased live birth and clinical pregnancy rates. The effect of LPS may be specifically present in protocols using gonadotropins for ovarian stimulation. However, due to the limited availability of data on the most clinically relevant outcomes – single-cycle and cumulative live birth rates – and the low to moderate study quality, the application of the synthesized evidence in daily clinical practice can clearly be questioned, and additional data from well-designed and adequately powered trials are required to validate or disprove our findings.

## Data availability statement

The original contributions presented in the study are included in the article/**Supplementary Material**. Further inquiries can be directed to the corresponding author.

## Author contributions

GC, TL, CL, FB, KD participated in the design of the paper. GC, TL, CL, KD performed the literature search, critical appraisal, and data extraction. GC, KD, and ME analyzed the data. GC and KD wrote the first version of the manuscript, supported by FB and AC. All authors were involved in the data interpretation, contributed to manuscript writing and approved the final version.

## Acknowledgments

The authors gratefully thank Paulien Wiersema from the Medical Library, UMC Utrecht for her contribution to the conceptualization and execution of the systematic search.

## Conflict of interest

FB is a Member of the external advisory board for Merck Serono and Ferring BV has received grants and personal fees outside the submitted work.

The remaining authors declare that the research was conducted in the absence of any commercial or financial relationships that could be construed as a potential conflict of interest.

## Publisher’s note

All claims expressed in this article are solely those of the authors and do not necessarily represent those of their affiliated organizations, or those of the publisher, the editors and the reviewers. Any product that may be evaluated in this article, or claim that may be made by its manufacturer, is not guaranteed or endorsed by the publisher.
