# Supplementary material for: The efficacy and safety of luteal phase support with progesterone following ovarian stimulation and intrauterine insemination: A systematic review and meta-analysis
Source: Front Endocrinol (Lausanne). 2022 Sep 2;13:960393. doi: 10.3389/fendo.2022.960393 (PMC9481250; doi:10.3389/fendo.2022.960393)

Supplementary figure 1. Funnel plots of the studies included in the syntheses of (**A**) cumulative live birth, and (**B**) cumulative clinical pregnancy. Data points represent the estimated risk ratio and associated standard error for each of the included studies. The dashed vertical line and triangular zone denote in each case the estimate from fixed-effect meta-analysis and its 95% confidence interval. In the absence of heterogeneity and non-reporting bias, 95% of results from individual studies are expected to lie within this region.

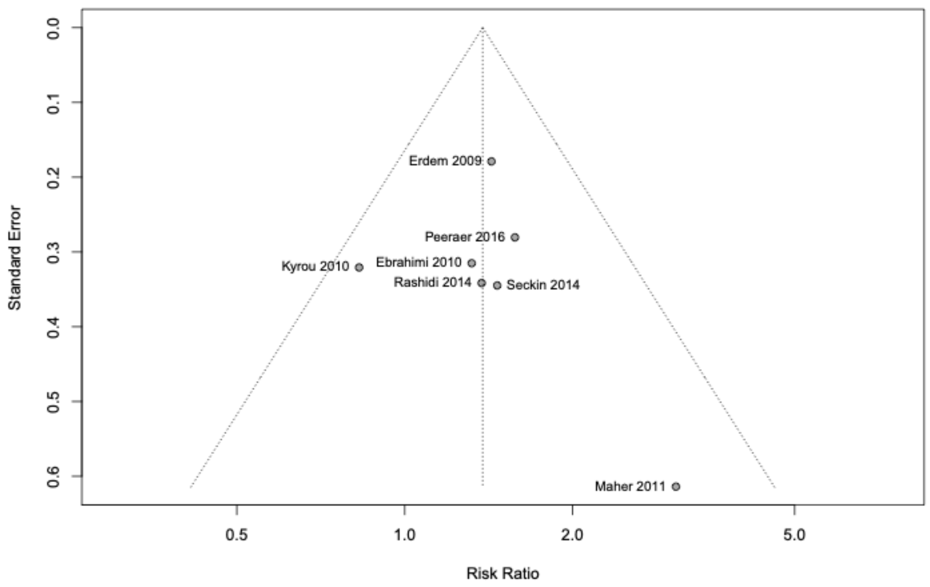

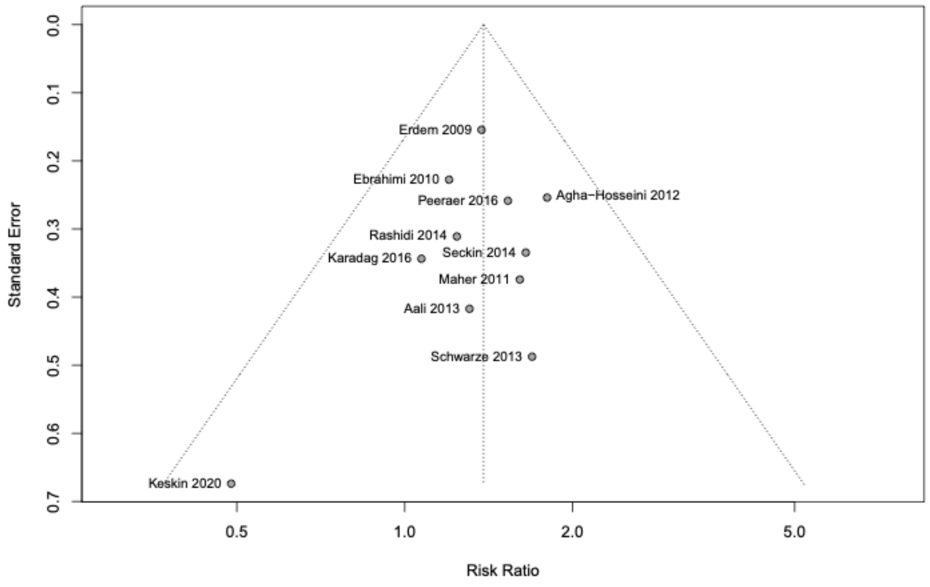

Supplementary figure 2. Forest plot of multiple pregnancy rate, cumulative over all study cycles. Comparison: progesterone luteal phase support versus placebo or no intervention. LPS: luteal phase support, CI: confidence interval.

Study	Experimental Events	Experimental Total	Control Events	Control Total	Weight	Odds Ratio Peto, Random, 95% CI
Agha–Hosseini 2012	3	36	2	20	17.6%	0.82 [0.12; 5.47]
Ebrahimi 2010	3	30	4	26	25.7%	0.62 [0.13; 2.97]
Erdem 2009	3	57	4	40	26.3%	0.50 [0.10; 2.35]
Karadag 2016	0	15	0	14	0.0%	
Kyrou 2010	1	17	2	19	11.7%	0.55 [0.05; 5.72]
Peeraer 2016	2	34	0	21	7.7%	5.20 [0.29; 92.54]
Schwarze 2013	0	9	0	6	0.0%	
Seckin 2014	2	18	1	12	11.1%	1.35 [0.12; 14.76]
**Total (95% CI)**		**216**		**158**	**100.0%**	**0.78 [0.38; 1.59]**
**Prediction interval**						**[0.25; 2.40]**

Heterogeneity: $Tau^2 = 0$; $Chi^2 = 2.36$, df = 5 (P = 0.80); $I^2 = 0\%$

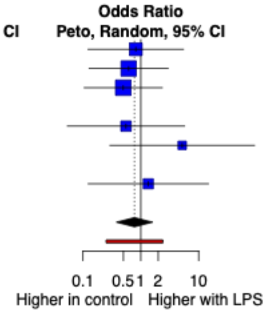

Supplementary figure 3. Miscarriage rate, cumulative. Forest plot of miscarriage rate, cumulative over all study cycles. Comparison: progesterone luteal phase support versus placebo or no intervention. LPS: luteal phase support, CI: confidence interval.

	Experimental		Control			Odds Ratio
Study	Events	Total	Events	Total	Weight	Peto, Random, 95% CI
Agha–Hosseini 2012	8	36	6	20	18.6%	0.67 [0.19; 2.32]
Ebrahimi 2010	9	30	7	26	22.0%	1.16 [0.37; 3.67]
Erdem 2009	8	57	7	40	23.6%	0.77 [0.25; 2.34]
Karadag 2016	1	15	0	14	1.9%	6.91 [0.14; 349.18]
Kyrou 2010	3	17	4	19	11.0%	0.81 [0.16; 4.13]
Peeraer 2016	4	34	3	21	11.1%	0.80 [0.16; 4.03]
Rashidi 2014	2	20	3	16	8.3%	0.49 [0.08; 3.20]
Schwarze 2013	0	9	0	6	0.0%	
Seckin 2014	2	18	0	12	3.5%	5.62 [0.32; 100.01]
**Total (95% CI)**		**236**		**174**	**100.0%**	**0.89 [0.56; 1.41]**
**Prediction interval**						**[0.45; 1.75]**

Heterogeneity: $Tau^2 = 0$; $Chi^2 = 3.52$, df = 7 (P = 0.83); $I^2 = 0\%$

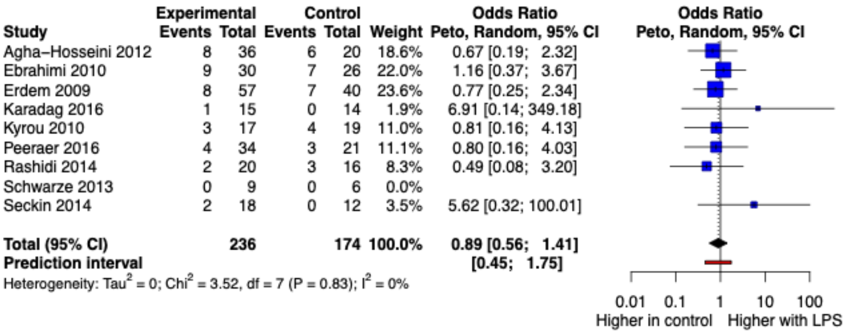

Supplementary figure 4. Univariate meta-regression analyses of the observed effect size versus progesterone dosage and duration of progesterone luteal phase support. **A.** and **B.** The plotted data points correspond to the risk ratio estimate for (**A**) cumulative live birth or (**B**) cumulative clinical pregnancy, and the applied dosage of progesterone in each individual trial. **C.** and **D.** The risk ratio estimates for (**C**) cumulative live birth or (**D**) cumulative clinical pregnancy are now plotted against the duration of progesterone luteal phase support in weeks since the onset of treatment (note that these are not gestational weeks). No significant correlations were observed. RR: risk ratio, LPS: luteal phase support.

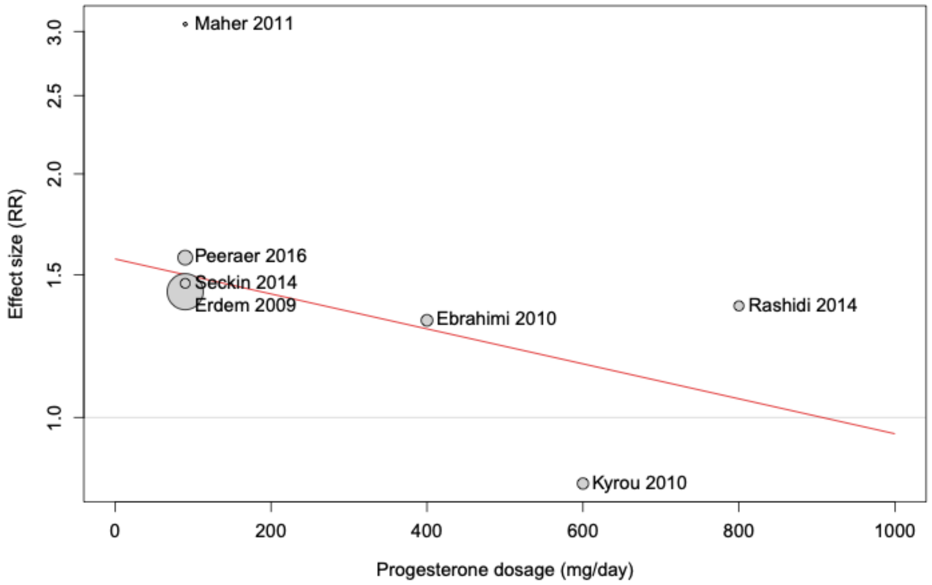

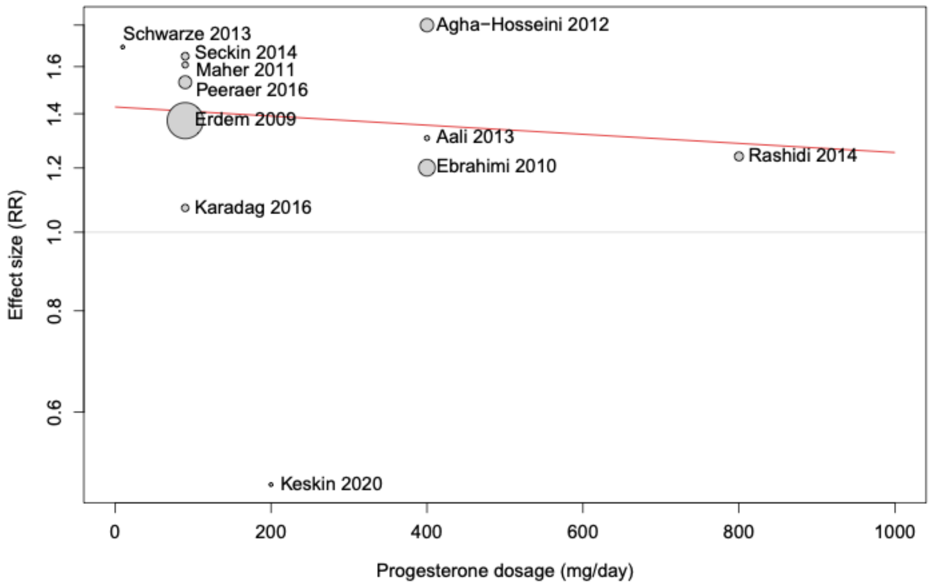

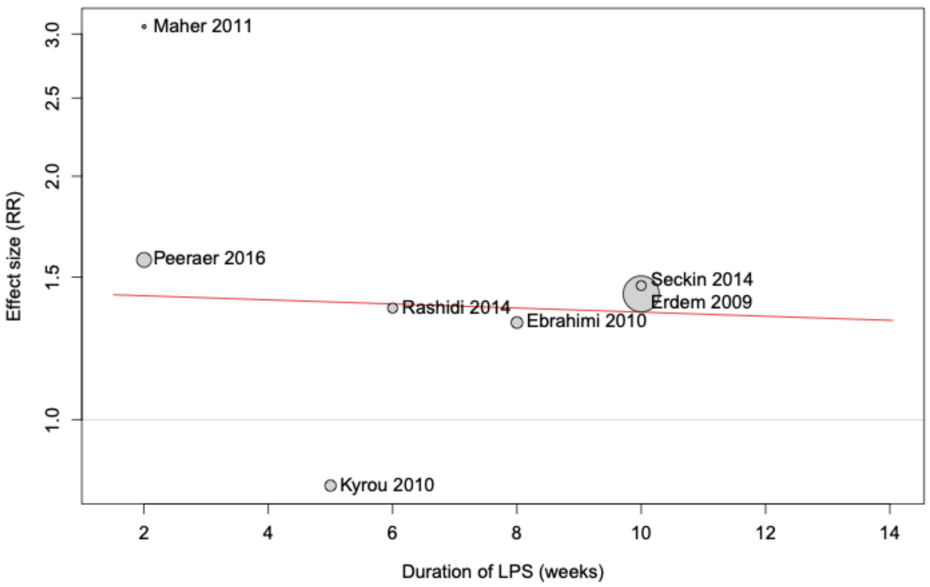
Maher 2011
Peeraer 2016
Seckin 2014
Erdem 2009
Rashidi 2014
Ebrahimi 2010
Kyrou 2010
Effect size (RR)
Duration of LPS (weeks)
3.0
2.5
2.0
1.5
1.0
2
4
6
8
10
12
14

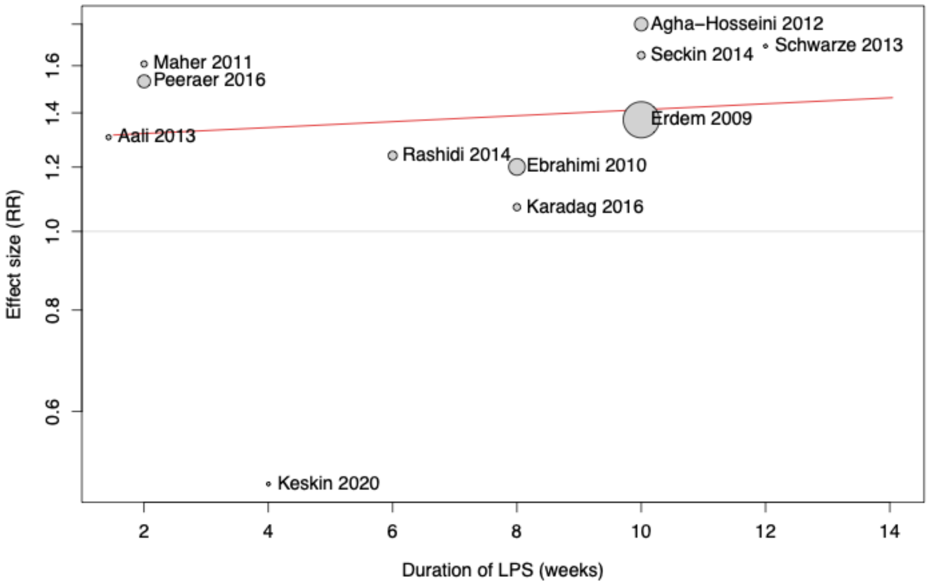
Agha–Hosseini 2012
Schwarze 2013
Seckin 2014
Maher 2011
Peeraer 2016
Erdem 2009
Aali 2013
Rashidi 2014
Ebrahimi 2010
Karadag 2016
Keskin 2020
Effect size (RR)
Duration of LPS (weeks)
1.6
1.4
1.2
1.0
0.8
0.6
2
4
6
8
10
12
14

Supplementary figure 5. Subgroup analysis by fraction of unexplained infertility. Comparison: progesterone luteal phase support versus placebo or no intervention in studies with a large fraction of participants diagnosed with unexplained infertility versus studies with mixed infertility diagnoses. **A.** Forest plot of live birth, cumulative over the whole study period. **B.** Forest plot of clinical pregnancy, cumulative over the whole study period. MH: Mantel-Haenszel, CI: confidence interval.

Study or Subgroup	Experimental Events	Experimental Total	Control Events	Control Total	Weight	Risk Ratio MH, Random, 95% CI
**Percentage unexplained ≤ 55%**						
Kyrou 2010	17	243	19	225	11.7%	0.83 [0.44; 1.55]
Maher 2011	10	37	3	34	3.2%	3.06 [0.92; 10.20]
Peeraer 2016	30	202	18	191	15.2%	1.58 [0.91; 2.73]
Rashidi 2014	18	127	13	126	10.3%	1.37 [0.70; 2.68]
**Total (95% CI)**		**609**		**576**	**40.4%**	**1.36 [0.66; 2.79]**
Heterogeneity: $Tau^2 = 0.0735$; $Chi^2 = 4.4$, df = 3 (P = 0.22); $I^2 = 32\%$						
**Percentage unexplained > 95%**						
Ebrahimi 2010	19	98	15	102	12.1%	1.32 [0.71; 2.44]
Erdem 2009	49	109	33	105	37.5%	1.43 [1.01; 2.03]
Seckin 2014	16	71	12	78	10.1%	1.46 [0.75; 2.88]
**Total (95% CI)**		**278**		**285**	**59.6%**	**1.41 [1.27; 1.58]**
Heterogeneity: $Tau^2 = 0$; $Chi^2 = 0.06$, df = 2 (P = 0.97); $I^2 = 0\%$						
**Total (95% CI)**		**887**		**861**	**100.0%**	**1.38 [1.09; 1.74]**
**Prediction interval**						**[1.04; 1.83]**
Heterogeneity: $Tau^2 = 0$; $Chi^2 = 4.53$, df = 6 (P = 0.61); $I^2 = 0\%$						
Test for subgroup differences: $Chi^2 = 0.03$, df = 1 (P = 0.87)						

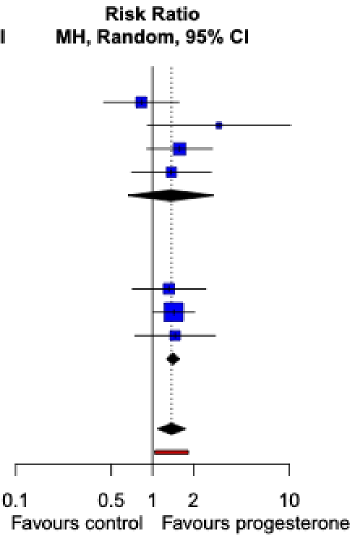

Study or Subgroup	Experimental Events	Experimental Total	Control Events	Control Total	Weight	Risk Ratio MH, Random, 95% CI
**Percentage unexplained ≤ 55%**						
Aali 2013	12	99	9	97	4.1%	1.31 [0.58; 2.96]
Agha−Hosseini 2012	36	150	20	150	11.2%	1.80 [1.09; 2.96]
Maher 2011	14	37	8	34	5.1%	1.61 [0.77; 3.35]
Peeraer 2016	34	202	21	191	10.8%	1.53 [0.92; 2.54]
Rashidi 2014	20	127	16	126	7.5%	1.24 [0.67; 2.28]
**Total (95% CI)**		**615**		**598**	**38.7%**	**1.52 [1.26; 1.85]**
Heterogeneity: $Tau^2 = 0$; $Chi^2 = 1.03$, df = 4 (P = 0.91); $I^2 = 0\%$						
**Percentage unexplained > 95%**						
Ebrahimi 2010	30	98	26	102	13.9%	1.20 [0.77; 1.88]
Erdem 2009	57	109	40	105	30.2%	1.37 [1.01; 1.86]
Karadag 2016	15	100	14	100	6.1%	1.07 [0.55; 2.10]
Keskin 2020	3	44	6	43	1.6%	0.49 [0.13; 1.83]
Schwarze 2013	9	47	6	53	3.0%	1.69 [0.65; 4.40]
Seckin 2014	18	71	12	78	6.4%	1.65 [0.86; 3.18]
**Total (95% CI)**		**469**		**481**	**61.3%**	**1.30 [1.03; 1.64]**
Heterogeneity: $Tau^2 = 0$; $Chi^2 = 3.46$, df = 5 (P = 0.63); $I^2 = 0\%$						
**Total (95% CI)**		**1084**		**1079**	**100.0%**	**1.38 [1.21; 1.59]**
**Prediction interval**						**[1.14; 1.68]**
Heterogeneity: $Tau^2 = 0$; $Chi^2 = 5.30$, df = 10 (P = 0.87); $I^2 = 0\%$						
Test for subgroup differences: $Chi^2 = 1.91$, df = 1 (P = 0.17)						

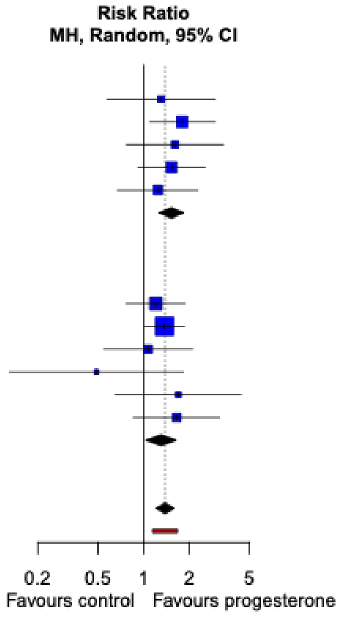

Supplement: Supplementary file 1 [file DataSheet_1.pdf]
