# Supplementary material for: The efficacy and safety of luteal phase support with progesterone following ovarian stimulation and intrauterine insemination: A systematic review and meta-analysis
Source: Front Endocrinol (Lausanne). 2022 Sep 2;13:960393. doi: 10.3389/fendo.2022.960393 (PMC9481250; doi:10.3389/fendo.2022.960393)
Supplement: Supplementary file 2 [file Table_1.docx]

**Supplementary table I.** Baseline participant characteristics and cycle characteristics of included studies.

| Study ID | %Unexplained infertility  [N (%)] | | Age in years [mean (SD)] | | BMI  [mean (SD)] | | Duration of infertility in years  [mean (SD)] | | Type of infertility  [N (%)] | | | | Day 3 FSH (IU/L)  [mean (SD)] | | peak E2 (pg/mL) [mean (SD)] | | # dominant follicles  [mean (SD)] | |
| --- | --- | --- | --- | --- | --- | --- | --- | --- | --- | --- | --- | --- | --- | --- | --- | --- | --- | --- |
|  |  |  |  |  |  |  |  |  | Primary | | Secondary | |  |  |  |  |  |  |
|  | P | control | P | control | P | control | P | control | P | control | P | control | P | control | P | control | P | control |
| Aali 2013 | 86 (44%) | 39 (39%) | 28 (NA) | 27.9 (NA) | NA | NA | 4.0 (NA) | 4.1 (NA) | 88 (89.9) | 86 (88.7) | 10 (10.1) | 11 (11.3) | 6.9 (2.4) | 7.2 (2.1) | NA | NA | NA | NA |
| Agha-Hosseini 2012 | 77 (52%) | 83 (59%) | 27.4 (3.7) | 26.8 (3.6) | 24.5 (3.0) | 24.0 (2.9) | 3.4 (1.3) | 3.2 (1.3) | NA | NA | NA | NA | 5.4 (3.1) | 5.1 (2.1) | NA | NA | 1.6 (0.7) | 1.4 (0.8) |
| Ebrahimi 2010 | 98 (100%) | 102 (100%) | 27.9 (3.3) | 28.4 (4.1) | NA | NA | 4.8 (2.6) | 4.84 (2.6) | 70 (71.4) | 71 (69.6) | 28 (28.6) | 31 (30.4) | 6.1 (2.7) | 6.7 (2.0) | NA | NA | 2.0 (0.8) | 2.2 (0.8) |
| Erdem 2009 | 109 (100%) | 105 (100%) | 30.0 (4.8) | 29.7 (4.3) | NA | NA | 4.7 (3.6) | 5.0 (3.3) | 70 (64.2) | 67 (63.8) | 39 (35.8) | 38 (36.2) | 7.3 (2.3) | 7.0 (2.7) | NA | NA | 1.6 (0.6) | 1.5 (0.9) |
| Karadag 2016 | 100 (100%) | 100 (100%) | 26.3 (4.2)^1^ 28.5 (3.6)^2^ | 25.7 (2.4) ^1^ 27.8 (3.7)^2^ | 24.5 (2.6) ^1^ 24.7 (2.4) ^2^ | 24.8 (2.6) ^1^ 23.6 (2.8) ^2^ | 2.6 (1.7) ^1^ 4.4 (2.5) ^2^ | 2.9 (1.9) ^1^ 4.7 (3.1) ^2^ | 33 (66) ^1^ 27 (54) ^2^ | 24 (48) ^1^ 29 (58) ^2^ | 17 (34) ^1^ 23 (46) ^2^ | 26 (52) ^1^ 21 (42) ^2^ | 6.4 (1.7) ^1^ 7.1 (4.2) ^2^ | 5.8 (1.6) ^1^ 6.8 (2.5) ^2^ | NA | NA | NA | NA |
| Keskin 2020 | 42 (95%) | 42 (98%) | 28 (4.1) | 26.7 (3.9) | 21.6 (2.8) | 22.1 (2.8) | 2.6 (3.5) | 2.8 (3) | NA | NA | NA | NA | 5.9 (NA) | 6.1 (NA) | NA | NA | 1.4 (0.6) | 1.1 (0.5) |
| Kyrou 2010 | 70 (36%) | 58 (29%) | 32.1 (3.6) | 32.2 (3.9) | 22.6 (2.8) | 22.4 (2.7) | A | NA | NA | NA | NA | NA | 7.2 (2.1) | 7.1 (1.9) | 513 (248) | 504 (306) | 1.2 (0.3) | 1.3 (0.4) |
| Maher 2011 | NA | NA | NA | NA | NA | NA | NA | NA | NA | NA | NA | NA | NA | NA | NA | NA | NA | NA |
| Peeraer 2016 | 56 (28%) | 55 (29%) | 31.5 (3.8) | 31.0 (4.0) | 23.0 (3.2) | 23.2 (3.4) | 2.4 (1.7) | 2.2 (1.6) | 120 (63) | 143 (71) | 70 (37) | 58 (29) | NA | NA | 311 (156) | 289 (157) | 1.2 (0.3) | 1.2 (0.3) |
| Rashidi 2014 | 33 (26%) | 31 (25%) | 27.5 (4.2) | 28.1 (4.6) | 22.7 (2.8) | 22.6 (2.6) | 4.6 (3.4) | 4.7 (3.3) | NA | NA | NA | NA | 6.0 (2.3) | 6.4 (2.6) | NA | NA | 2.2 (1.3) | 2.4 (1.3) |
| Schwarze 2013 | 47 (100%) | 53 (100%) | 31.5 (3.0) | 32.3 (3.1) | NA | NA | 3.8 (3.1) | 4.8 (3.1) | NA | NA | NA | NA | NA | NA | NA | NA | 2.0 (1.0) | 2.0 (1.0) |
| Seckin 2014 | 71 (100%) | 78 (100%) | 27.8 (5.2) | 28.9 (5.5) | NA | NA | 4.6 (3.0) | 4.6 (3.4) | 63 (88.7) | 8 (11.3) | 75 (96.2) | 3 (3.8) | 6.7 (2.2) | 6.8 (2.3) | 598 (323) | 670 (435) | 2.0 (1.3) | 2.3 (1.8) |

^1^Clomiphene Citrate stimulated subgroup, ^2^Gonadotropin stimulated subgroup

SD; standard deviation, P; progesterone, BMI; body-mass index.

**Title:** The effectivity and safety of luteal phase support with progesterone following mild ovarian stimulation and intra uterine insemination: A systematic review and meta-analysis

**Authors:** G Casarramona, T Lalmahomed, C Lemmen, MJC Eijkemans, AEP Cantineau, FJM Broekmans, KCE Drechsel

**CORRESPONDING AUTHOR**

Drs. K.C.E.Drechsel, MD

Department of Reproductive Medicine, University Medical Center Utrecht, Heidelberglaan 100, PO Box 85500, 3508 GA

Utrecht, The Netherlands

[k.c.e.drechsel-2@umcutrecht.nl](mailto:k.c.e.drechsel-2@umcutrecht.nl)
